# Pathophysiology of refractory obsessive-compulsive disorder: A study of visual search combined with overactive performance monitoring

**DOI:** 10.1097/MD.0000000000005655

**Published:** 2017-01-10

**Authors:** Qingxiao Liu, Bo Tan, Jing Zhou, Zhong Zheng, Ling Li, Yanchun Yang

**Affiliations:** a Mental Health Centre, West China Hospital, Sichuan University; b Key Laboratory for Neuroinformation of the Ministry of Education, School of Life Science and Technology, University of Electronic Science and Technology of China, Chengdu, China.

**Keywords:** dorsolateral prefrontal cortex, event-related potentials, eye movement, fusiform gyrus, obsessive–compulsive disorder, refractory

## Abstract

Based on both functional and structural studies of excessive activity, fronto-striatal-thalamic-cortical and cortico-striatal circuits have been hypothesized to underlie the pathophysiology of obsessive-compulsive disorder (OCD). However, the neurobiological underpinnings of OCD refractory to medication and therapy remain controversial. This study aimed to evaluate neuroanatomical abnormalities of the whole brain and to evaluate visual processing in patients with refractory OCD.

This study was comprised of 2 experiments. The neuroanatomical abnormalities of the whole brain were evaluated using a visual search in combination with overactive performance monitoring (Experiment I), and visual processing was evaluated using event-related potentials recorded from subjects during performance of a visual search task. We also examined the amplitudes and latency of the error-related negativity (ERN) using a modified flanker task (Experiment II). Standard low-resolution electromagnetic tomography analysis was applied to determine the special areas.

Patients with refractory OCD had a significantly greater number of saccades and prolonged latencies relative to the healthy controls. Scalp map topography confirmed that visual cognitive and executive dysfunction was localized to the fusiform gyrus. Furthermore, we found that during a modified flanker task, ERNs had a greater amplitude and a prolonged latency relative to those of the healthy controls. Further data analysis suggested that cognitive dysfunction and compulsive behavior in OCD patients were linked to abnormalities within the dorsolateral prefrontal cortex (DLPFC).

We identified abnormal activities within the fusiform gyrus and DLPFC that likely play important roles in the pathophysiology of OCD.

## 1 Introduction

Obsessive-compulsive disorder (OCD) is an anxiety disorder characterized by the presence of intrusive thoughts as well as repetitive and ritualistic behaviors. OCD is associated with significant functional impairments and interference of the quality of life. Worldwide, lifetime prevalence estimates for OCD are approximately 2%.^[1]^ Two popular and well-characterized treatments for OCD are cognitive behavior therapy and selective serotonin reuptake inhibitors, with 40% to 60% of patients achieving significant symptomatic improvement.^[2]^ However, approximately 10% of patients exhibit no improvement despite long-term treatment with various therapies and are considered to have severe, treatment refractory OCD.^[3]^

To date, research has focused on understanding the brain function abnormalities that contribute to the pathophysiology of OCD.^[4]^ Data from functional magnetic resonance imaging studies have revealed the importance of some key brain regions, including the frontal cortex, orbitofrontal cortex (OFC), paralimbic system (anterior cingulate cortex [ACC], temporal cortex, and insular cortex), limbic system, striatum, and thalamus.^[5]^ The measurement of event-related potential (ERP) directly reflects neural activity associated with sensory, cognition, and motor events; moreover, ERPs are sensitive to both the extent (amplitude) and speed (latency) of processing during the cognitive stages under evaluation.^[6]^ Detection of ERPs by electroencephalography (EEG) has identified a cluster of modules in the medial frontal cortex that may be involved in overactive performance monitoring in OCD.^[7]^ Based on the topographic distribution of EEG activity, the source of error-related negativities (ERNs) can be inferred, yielding the generation of 2 main hypotheses.^[8]^

In one model proposed by Pitman, excessive error signals generated by the ACC lead to alerted cognitive, motor, and affective systems that promote the need to correct the problem and the generation of compulsive behaviors.^[9,10]^ Another model predicts that OCD is associated with OFC hyperactivity. The medial OFC is extensively connected to the limbic system and diencephalon and is involved in motor control and stimulus-response learning. The lateral OFC plays a role in monitoring inhibitory and excitatory regulation of behavior and emotion via its connections with the dorsolateral prefrontal cortex (DLPFC). Both parts of the OFC project to the striatum in the basal ganglia, and excessive activation of the cortico-basal ganglia-thalamocortical loops likely mediates the symptomatology of OCD.^[11]^

At present, few studies have investigated the pathophysiology of refractory OCD. There are some differences in the structural imaging between refractory OCD and responding OCD. Refractory patients have significantly greater left and right thalamus volumes compared with responding patients and healthy controls.^[12]^ Moreover, refractory OCD patients, but not responding OCD patients, have shown statistically significant smaller hippocampal and amygdala volumes relative to healthy controls.^[13]^ A promising and reversible neuromodulatory treatment for refractory OCD is anterior capsular stimulation, which causes a decrease in the metabolic activity of the prefrontal cortex, especially in the subgenual ACC.^[14]^ It has been reported that the subgenual ACC and ventral striatum are key players in the pathological neuronal circuits responsible for refractory OCD.^[15]^ Deep brain stimulation treatment in the ventral caudate nucleus may increase the activity of the ACC region and improve the symptoms of OCD.^[14,15]^ These findings suggest that abnormal activity of the subgenual ACC may contribute to refractory OCD.

Neuropsychological studies of OCD patients have documented various impairments in a number of cognitive domains, including nonverbal memory, visuospatial skill, visual attention, and selective attention.^[16]^ Although cognitive dysfunction has been associated with various abnormalities in brain regions,^[17–19]^ it is unclear how these changes translate into the cognitive impairments of refractory OCD seen clinically. To further study brain dysfunction and impairment, a commonly used cognitive operation is the visual search, a perceptual task that requires high flexibility for the representation of targets.^[17]^ In the visual search, there are multiple items considered to be targets as well as some heterogeneous items to be distractors. By reducing distractor heterogeneity via grouping, the search task for categorically defined targets can be facilitated.^[18]^ It has been reported that categorical guidance may be weak or nonexistent for search tasks using common realistic object categories.^[19]^ Herein, we modified the visual based flanker interference task by increasing the complexity and presenting realistic object targets to enhance the reliability and accuracy of our study.

Overactive performance monitoring by examining persistent ERPs represents an electrophysiological correlate of OCD. The ERN is a specific component of the ERP that was initially identified following execution of an incorrect response.^[20]^ Using dipole source localization and functional imaging techniques based on quantitative 3-dimensional presentation of EEG, the brain regions responsible for generating ERN were determined.^[21]^ The single-trial ERN amplitudes are enhanced in OCD patients, and this abnormal activity may reflect cognitive impairment.^[22]^ Cavanagh et al^[23]^ have provided a new method by analyzing evidence from these imaging and anatomical studies to investigate the mechanism for understanding the role of brain regions in behavior and cognitive control.

The aims of the current study were to investigate cognitive activity related to OCD by a combination of data from eye movement and ERPs, which is achieved with a visual search task combined with an ERP experiment, and to evaluate cognitive impairments in OCD patients by monitoring ERNs under overactive performance using a modified flanker interference task.

## 2 Patients and methods

This study was comprised of two experiments. Experiment I included a visual search combined with an ERP experiment, and Experiment II was carried out using the modified flanker interference task.

### 2.1 Subjects

The 2 studies (I and II) were conducted in accordance with the Declaration of Helsinki, and the study protocol was approved by the Ethics Committee of West China Hospital of Sichuan University (Chengdu, China). Written informed consent was obtained from all participants.

OCD patients were recruited to the study from among all the outpatients and inpatients of the Mental Health Center of West China Hospital who were diagnosed with OCD according to the Diagnostic and Statistical Manual of Mental Disorders (DSM-IV) criteria.^[24]^ Patients were included if they exhibited the following criteria: aged from 25 to 45 years, were right-handed, and had a Yale Brown Obsessive Compulsive Scale (Y-BOCS)^[25]^ score of at least 16. Patients were excluded if they exhibited any of the following criteria: brain organic psychosis, neurological impairment, severe endocrine disease, or metabolic disorder, traumatic brain injury with coma, vomiting, or amnesia syndrome, traumatic eye injury, a history of extraocular muscle disease, or myopia over 400 degrees, syphilis or autoimmune immunodeficiency syndrome, addiction to alcohol, or other psychiatric disorder diseases (ie, schizophrenia, mood disorders, or substance-related disorders).

Distinct OCD psychopathology was specifically assessed with the Y-BOCS, and further symptomatic assessment was obtained with the Obsessive-Compulsive Inventory, Revised (OCI-R).^[26]^ All participants self-reported the level of depression using the Beck-Depression-Inventory (BDI), with a threshold score of 5 in the Chinese version (1994).

All enrolled OCD patients showed similar Y-BOCS and OCI-R scores at baseline. To investigate the pathological mechanism underlying refractory OCD, 2 well-defined and selected subgroups were established for comparative analysis at the end of the follow-up. The refractory OCD patients had the following characteristics: OCD for >3 years; a total Y-BOCS score >25 or an individual Y-BOCS score >15; improvement of the Y-BOCS score of <25% or a less than minimal improvement on the Clinical Global Impressions (CGI) scale after >2 years of adequate pharmacotherapy involving selective serotonin reuptake inhibitors plus clomipramine at the maximum recommended dosage for 12 weeks in addition to combination treatment with atypical antipsychotic drugs for 4 weeks; nonresponse to at least 20 hours of ≥1 adequate sessions of cognitive behavioral therapy. Responding patients were selected from among the entire population of OCD patients who had undergone and completed treatment with any conventional therapy and who had demonstrated ≥50% reduction in the Y–BOCS score or on the CGI scale, as compared to baseline, and for which the change had been maintained for at least 1 year.

### 2.2 Performance

#### 2.2.1 Experiment I: Visual search task

This task was conducted with a program written by E-Prime 2.0 (Psychology Software Tools, Inc., Sharpsburg, PA). The program consisted of 300 subtrials, each of which was set to last between 2600 and 4100 ms. Every 60 subtrials formed 1 unit, and trial subjects were allowed a short rest after every unit. The first unit was applied as a practice session, and the subjects were allowed to repeat the entire unit as many times as they felt necessary to gain a comfortable mastery; the data obtained from this practice unit were excluded from the statistical analysis.

Images from a total of 6 categories (each containing 40 images of daily-life objects) were selected as the targets, including vehicles, kitchen appliances, small animals, musical instruments, fruits and vegetables, and small household appliances. For each test, images from only 1 category were set as targets. Interference images are images containing many objects that are messily set out and unclean.

**Figure 1:**
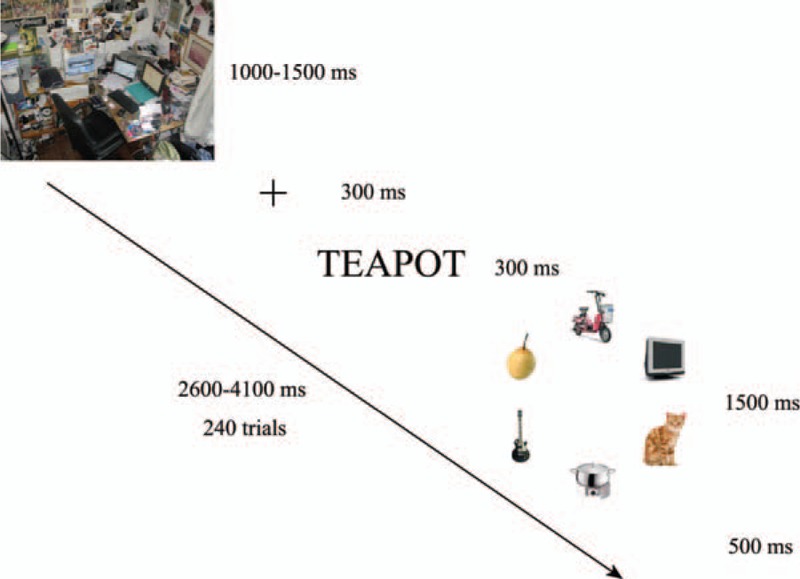
Experimental design of the visual search task (Experiment I).

As shown in Figure 1, for each trial, the computer screen displayed or did not display an interference image first. Then, the interference images disappeared, and a fixation marker “+” and a word referring to the target appeared in a neat and orderly manner in the center of the screen. Next, one object image from one category appeared. Finally, 6 object images from the 6 categories, respectively, including the target image or not, were presented in the center of the screen with a 2.4-degree visual angle radius. The object images were presented in square borders with a 2-degree visual angle on each side. The 12 o’clock position site was considered as the start point, and the 6 object images were randomly distributed in the circumferential position of visual angles (0, 60, 120, 180, 240, and 300 degree). The screen resolution was 1024 × 768. The subjects sat in front of the screen at an average distance of 61 cm. They were asked to look at the center of the screen and to respond within 1500 ms whether or not the object image referred to the word that appeared on the screen; their answers were recorded by a right-hand click on a mouse using the left button for “YES” and the right button for “NO.” Each subject was required to perform 2 units with interference images and 2 units without interference images, alternately.

#### 2.2.2 Experiment II: Modified flanker interference task

**Figure 2:**
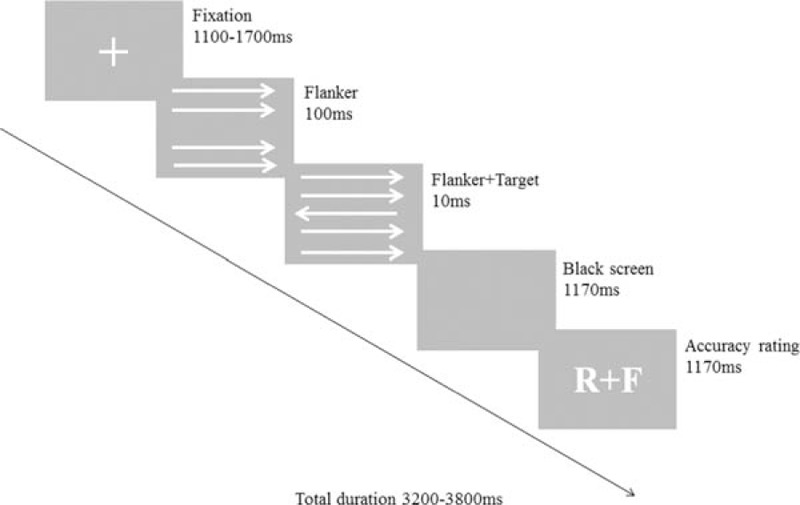
Experimental design of the modified flanker task (Experiment II). Subjects were instructed to respond by clicking the right or left mouse button to indicate the target arrow direction. Subsequently, the subjects rated their previous response as correct, incorrect, or unsure. The letters R and F refer to correct (“right”) and incorrect (“false”), respectively.

The modified flanker interference task was modified based on the design reported previously.^[6]^ Each experiment was composed of 420 subtrials, and every trial lasted for 3200 to 3800 ms. The target image was composed of 5 parallel arrows, which were 2.4 degree tall and 0.5 degree wide. The subjects were required to identify the direction of the middle target arrow while ignoring other arrows. The first 60 trials were a practice unit for the subjects to practice and master this task. Data obtained from this unit were excluded from the final statistical analysis. The remaining 360 trials were divided equally into 2 units, and the subjects rested after every unit. As shown in Figure 2, for each trial, a fixation mark “+” was first presented at the center of the screen for 1100 to 1700 ms, then 4 horizontal flanker arrows pointing either left or right appeared for 100 ms (Fig. 2). The middle fifth target arrow then appeared pointing to the left or right (one-third of the arrows were in the same direction as the other 4 arrows) for 10 ms. Next, the screen was left blank for 1170 ms. The subjects were required to indicate the direction of the target arrow during the time from the target arrow appearing until the blank screen disappeared.

#### 2.2.3 Data collection and analysis

The mean response time and accuracy rate were recorded in the 2 experiments, and the subjects were divided into 4 subgroups in Experiment I: objective without interference (S1); no objective and no interference (S2); objective with interference (S3); no objective with interference (S4).

Eye tracking data were recorded using the EyeLink 1000 Multiple Eye Tracking Solutions in One system (SR Research Ltd, Ottawa, Canada) and processed using Matlab 7.0 software (MathWorks, Natick, MA) to obtain the mean number of saccades (sac #; value: n), saccade latency (SL; ms), saccade amplitude (sacAmp; RMS), saccade duration (sacDur; ms), and fixation duration (FixDur; ms). Ocular correction was performed offline utilizing the algorithm of Gratton and Coles.^[27]^ To account for (and reject) artifacts, trials were excluded if the voltage had exceeded ±70 μV.

Data from a 64-channel, 64-electrode BrainCap MR-compatible EEG cap (Brain Products, Gilching, Germany) were captured and digitized at 500 Hz. Additional electrodes were positioned above and below the left eye, on the outer canthi of each eye, and on each mastoid.

BrainVision Analyzer 2.01 (Brain Products, Germany) classified the ERP data and performed automated correction for ocular artifacts, followed by low-pass filtering to 50 Hz and recalculation using the TP9 and TP10 mastoid sites as references. The resultant data were transformed for complementary analysis in Matlab 7.0. For Experiment I, the behavior data were designated as 1 of 2 response types (correct or error). The ERP data of “correct” responses were averaged. The amplitudes of effective components and the corresponding latency of the ERP data in the 4 groups were analyzed. For Experiment II, the ERN or the error negativity (Ne) was quantified using a minimum of between 6 and 8 error trials, and the criterion for obtaining an EEG artifact-free trial of error response was >7. The ERN data were averaged synchronously with time-locked response onset (200 ms pre-onset and 800 ms post-onset). The ERN amplitude was determined for the peak-to-peak differences, which were calculated by subtracting the amplitude of the relatively positive peak that immediately preceded the ERN from the negative peak. The positive peak was searched for from among the data taken at −100 to 0 ms preceding the response onset; the negative peak was searched for among the data taken in the time frame but from 0 to 150 ms following the response onset. Peak latencies were determined at FCz. The amplitudes and the corresponding latency of the ERN data at 5 electrodes (FPz, Fz, FC1, FC2, and Cz) from the frontal lobe to the parietal lobe were analyzed.

#### 2.2.4 Standard low-resolution electromagnetic tomography analysis

After affirming intergroup differences in ERPs, the location(s) of the related brain generators were determined to provide insights into the cognitive changes that occurred across conditions. The scalp map was applied to the data obtained from the 64 cap electrodes (located on the right-fitting sphere), and the scalp topography was mapped by computing using the standard low-resolution electromagnetic tomography (sLORETA) software to generate a 3-dimensional map representation of the data. The sLORETA software package is available online at: http://www.uzhch/keyinst/loreta. This calculation of the standardized current source density is based on the collected scalp activity. In the sLORETA analysis, the cortex is modeled at 6239 voxels in the gray matter, and the hippocampus and amygdala are modeled using the digitized Montreal Neurological Institute coordinates corrected to the Talairach coordinates. By this method, images for each ERP in the experiments were constructed to present the given moment when the maximum density was taken as well as the source of the particular component. The current density magnitudes in 3 dimensions (sLORETA-xyz values) were computed with paired and independent *t* tests to determine the differences between conditions and groups with normalization, respectively.^[28]^ The activation of a particular Broadmann area was projected onto the sLORETA analysis.

#### 2.2.5 Statistical analysis

Statistical analyses were performed using SPSS 16.0 (SPSS Inc. Chicago, IL). All data are presented as mean ± standard deviation. Comparisons between groups were performed using the *χ*^2^ test (categorical variables) or the *t* test (continuous variables). Analysis of variance with the post-hoc Turkey test was used when comparing 3 groups. Repeated measures were compared using a paired *t* test. A *P* value <0.05 was considered statistically significant.

## 3 Results

### 3.1 Characteristics of the subjects

For the combination visual search and ERP experiment (Experiment I), 21 OCD patients (13 responding and 8 refractory) and 13 healthy subjects were enrolled. For the overactive performance monitoring experiment (Experiment II), 18 OCD patients (13 responding and 9 refractory) and 12 healthy subjects were enrolled. There was no overlap in the participants between the 2 experiments. As shown in Table 1, the OCD patients and healthy controls did not differ in sex, age, education, or verbal intelligence. There was no statistically significant difference in the Y-BOCS or OCI-R scale scores between the responding and refractory OCD patients at baseline.

**Table 1:**
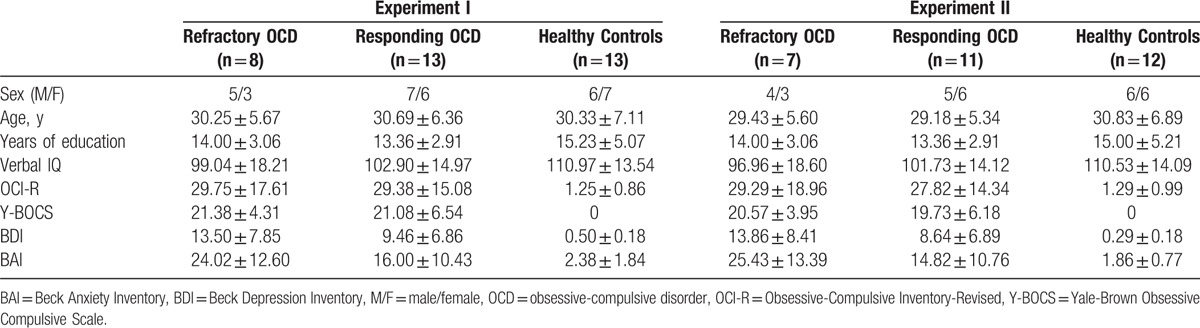
Demographic and psychopathological characteristics of OCD patient subgroups and healthy control subjects.

### 3.2 Results of the visual search (Experiment I)

#### 3.2.1 Behavioral data

The response time in the healthy controls was significantly less than that in both the responding and refractory OCD patients (*P* < 0.001) (Table 2). Regarding overall accuracy, there was no statistically significant difference between the healthy controls and the responding subgroup, whereas the control and responding OCD patients exhibited a higher accuracy rate than the refractory subgroup (*P* < 0.005). Without interference, the refractory subgroup had a higher error response rate than the healthy controls and the responding subgroup. With interference, the refractory subgroup exhibited a greater slowing in the post-error response time.

**Table 2:**
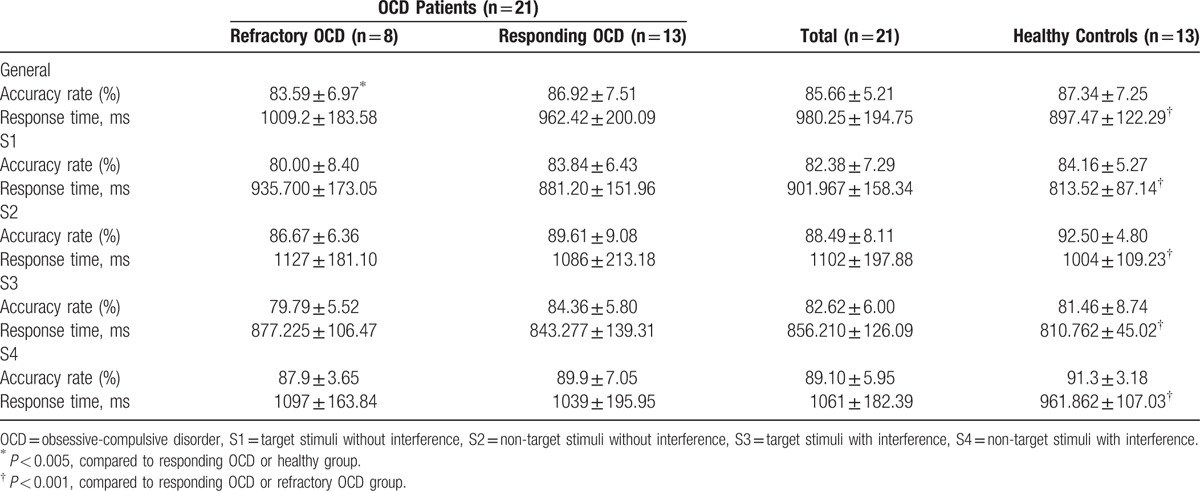
Behavioral characteristics of OCD patient subgroups and healthy control subjects in the visual search combined with ERP (Experiment I).

#### 3.2.2 Eye movement data

Considering eye movements, compared to patients with OCD, the healthy controls performed a greater number of saccades (2.98 ± 0.65 vs. 3.20 ± 0.88, *P* = 0.038) and exhibited a reduction in the saccade latency (38.47 ± 3.17 vs. 36.60 ± 3.14, *P* < 0.001) as well as a decrease in the fixation duration (206 ± 21.06 vs. 216.38 ± 28.27, *P* = 0.029) (Table 3). When comparing the subgroups, the healthy controls had a significantly greater number of saccades than the refractory subgroup (2.98 ± 0.65 vs. 3.29 ± 0.81, *P* = 0.029), but there was no statistical difference between the healthy controls and the responding subgroup. Patients with responding OCD showed an extended fixation duration compared to patients with refractory OCD (37.08 ± 2.98 vs. 35.82 ± 3.27, *P* = 0.012) (Table 3).

**Table 3:**
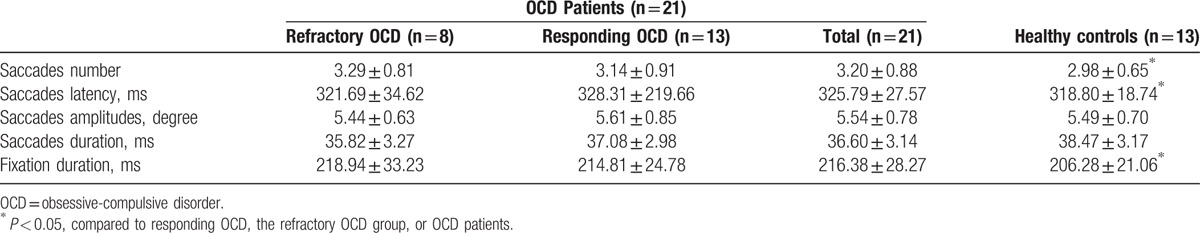
Eye movements of OCD patient subgroups and healthy control subjects (Experiment I).

#### 3.2.3 ERP components

**Figure 3:**
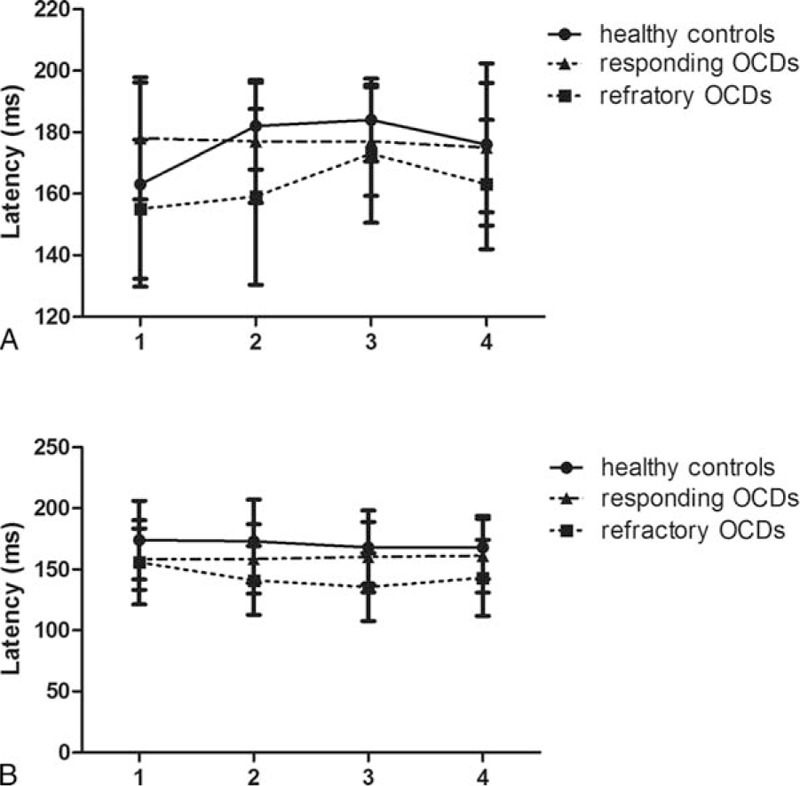
The latency (ms) of component 1 at electrodes C3 (A) and C4 (B) in healthy controls, responding OCD patients, and refractory OCD patients (Experiment I). OCD = obsessive-compulsive disorder.

Two major ERP components (components 1 and 2) related to eye movements were typically observed in Experiment I. Component 1, the latency of which ranged from 120 to 200 ms after target onset, was similar to N1, whereas it appeared as a positive polarity in the frontal lobe and with opposite polarity in the occipital lobe. The latency of component 1 showed some difference between groups. Although no difference was observed between the healthy controls and the OCD patients, a difference was observed between the refractory OCD subgroup and the healthy controls as well as between the refractory OCD subgroup and the responding OCD subgroup. The characteristic differences were mainly located in electrodes C3 and C4. At electrode C3, the refractory subgroup in the S2 situation exhibited earlier peak latencies relative to those of the healthy controls (*P* = 0.04; Fig. 3A). At electrode C4, the refractory subgroup in the S2 and S3 situations exhibited delayed peak latencies compared to those of the healthy controls (*P* = 0.047, *P* = 0.043, respectively; Fig. 3B).

Component 2, the latency of which ranged from 250 to 300 ms after target onset, was similar to N2 or P3a, whereas it appeared as a negative polarity in the frontal lobe and with opposite polarity in the occipital lobe.

**Figure 4:**
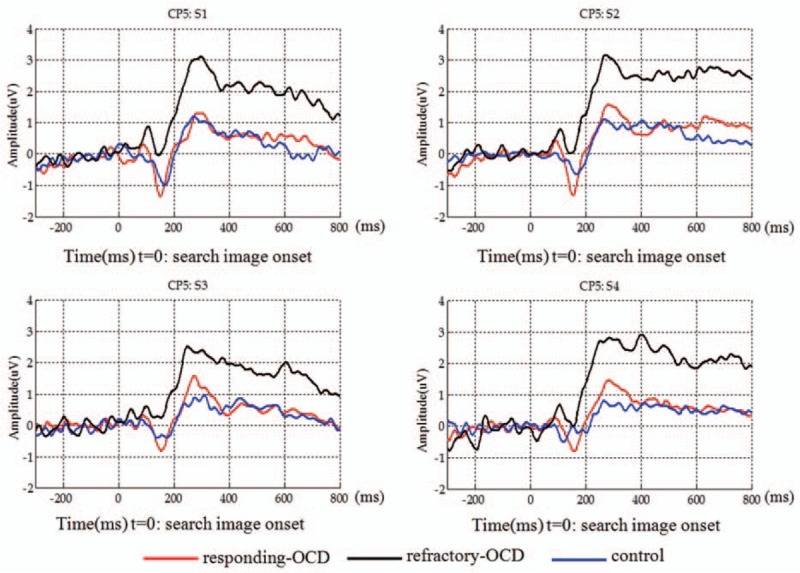
Averaged event-related potential waveforms of component 2 at electrode CP5 of the OCD patient subgroups and the healthy controls (Experiment I). OCD = obsessive-compulsive disorder.

The difference for component 2 was observed in the peak amplitudes between different groups. The difference was mainly located in the occipital lobe with distribution mostly outside of the left hemisphere, ranging from electrodes CP5 and TP7 to O1 and Oz. Representative pictures are shown for electrode CP5: the healthy controls exhibited higher peak amplitudes in S1 to S3 situations relative to those of the responding and the refractory OCD subgroups (*P* = 0.013, *P* = 0.029 in S1, *P* = 0.051, *P* = 0.048 in S2, *P* = 0.008, *P* = 0.044 in S3, respectively), and higher peak amplitudes in S4 situations relative to those of the refractory OCD subgroup (*P* = 0.011) (Fig. 4).

**Figure 5:**
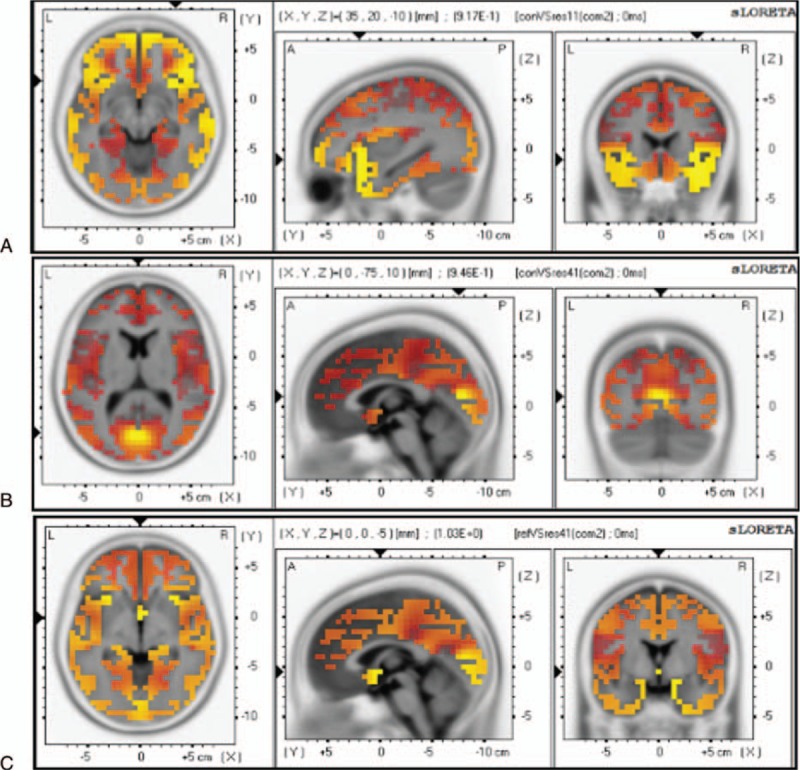
sLORETA images focusing on the largest difference observed between the healthy controls and the OCD patients in the visual search (Experiment I). Yellow-Red indicates the activated areas between the two groups and presents the voxel with the most significant sLORETA values. (A) Inferior frontal gyrus (x, y, z = 35, 20, −10; BA = 47). (B) Occipital lobe (x, y, z = 0, −75, 10; BA = 18). (C) Anterior cingulate (x, y, z = 0, 0, −5; BA = 25). *P* < 0.05. OCD = obsessive-compulsive disorder, sLORETA = standard low-resolution electromagnetic tomography.

Figure 5 illustrates the scalp ERN topographies after commission of error responses for components 1 and 2, respectively. Although activation can be observed in many regions, including the insula, operculum, and parietal cortex, statistical comparative analysis of the control group and the OCD patient group showed no significant differences for component 1, but did show a significant difference (decrease in the amplitudes) for component 2, especially in the frontal lobe, occipital lobe, and parietal lobe, and sometimes in the temporal lobe. Thus, component 2 will generate in an area between the interior frontal gyrus and the occipital lobe, particularly in the fusiform gyrus (Fig. 5A and B and Table 4). When correlation analysis was performed, a relationship was detected for ACC, with differences observed between the refractory and responding OCD patients (Fig. 5C and Table 4).

**Table 4:**
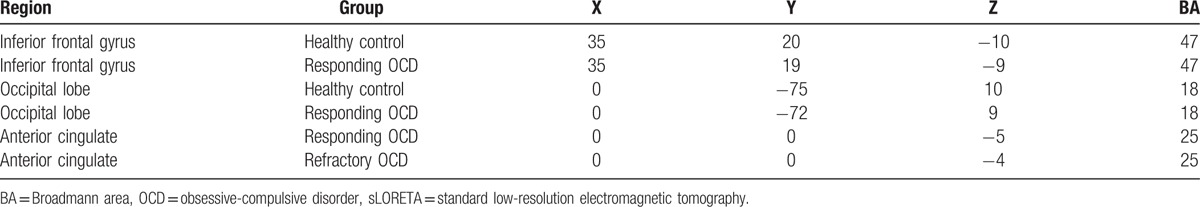
Differences between the healthy controls and the OCD patients preceded in sLORETA in the visual search task (Experiment I).

### 3.3 Results from the modified flanker interference task (Experiment II)

#### 3.3.1 Behavioral data

The error rates and reaction times of all participants are shown in Table 5. Compared to the healthy controls, patients with OCD had a significantly lower accuracy rate (63.89% vs. 87.6%, *P* = 0.02) and showed more errors (94.33 vs. 65.91 ms, *P* = 0.07) and a longer correct reaction time (378 vs. 424 ms, *P* = 0.001). Compared to the responding OCD subjects and the heathy controls, the refractory OCD subjects showed a lower accuracy rate, more errors, a shorter correct response time, and a longer error response time (all *P* < 0.05, Table 5).

**Table 5:**
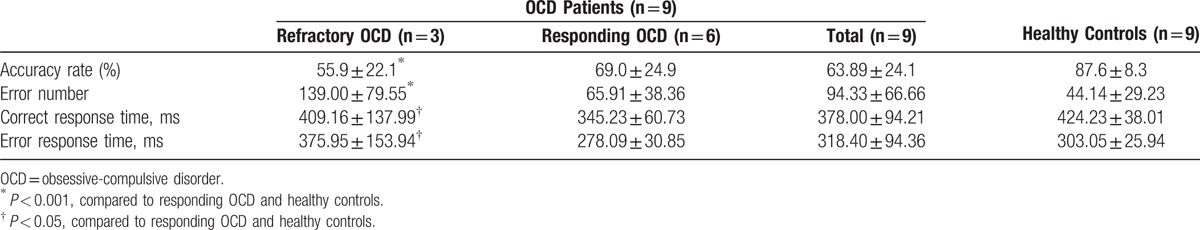
Behavior data of OCD patient subgroups and healthy control subjects in the modified flanker interference task (Experiment II).

#### 3.3.2 ERN data

**Figure 6:**
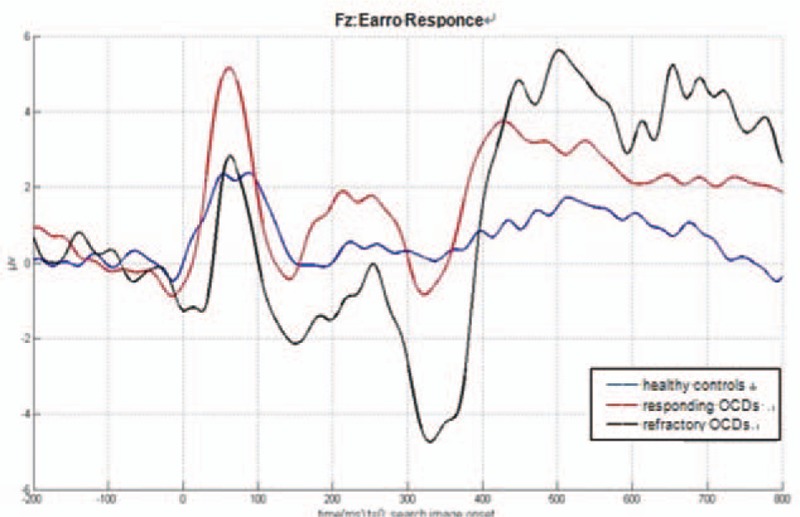
Averaged event-related potential waveforms at electrode Fz of the OCD patient subgroups and the healthy controls (Experiment II). Significantly decreased ERN amplitudes were observed in the refractory OCD patients compared to the healthy controls (*P* < 0.005 by latency analysis). ERN = error-related negativity, OCD = obsessive-compulsive disorder.

The average ERN amplitudes and latencies of all participants at 5 selected electrodes (FPz, Fz, FC1, FC2, and Cz) from the frontal lobe to the parietal lobe are shown in Table 6. Compared to the control group, the OCD patient group showed reduced ERN amplitudes at the Fz, FC1, FC2, and Cz electrodes. The Fpz electrode showed significantly lower ERN amplitudes for the refractory OCD subgroup, whereas it showed higher ERN amplitudes for the responding OCD subgroup, compared to the healthy controls. In addition, analysis of the differences between the ERN amplitudes indicated an interaction between groups and electrodes. The most significant difference was found for Fz in the refractory OCD patients, compared to the healthy controls (*P* < 0.005; Fig. 6). In addition, greater latencies were observed for the OCD patients, compared to the healthy controls, at all electrodes except for Fpz (Table 6).

**Table 6:**
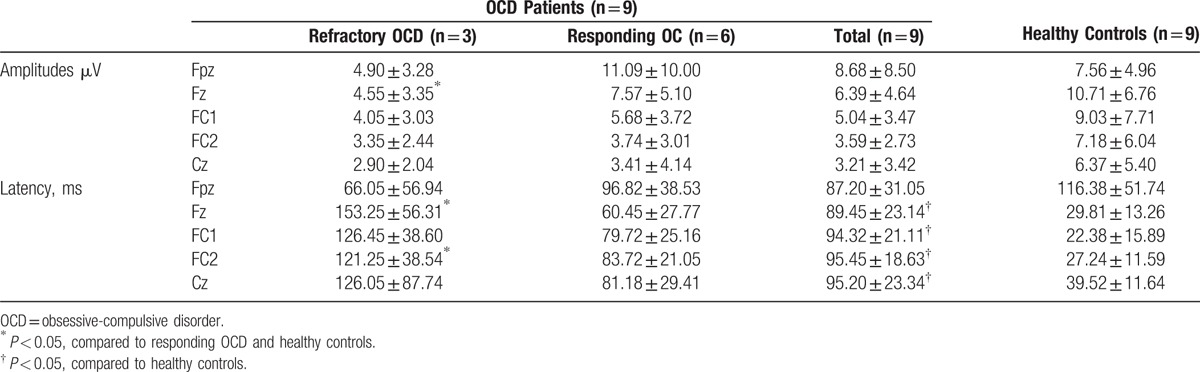
The ERN amplitudes and latency at five selected electrodes from the prefrontal lobe to the parietal lobe in the modified flanker interference task (Experiment II).

**Figure 7:**
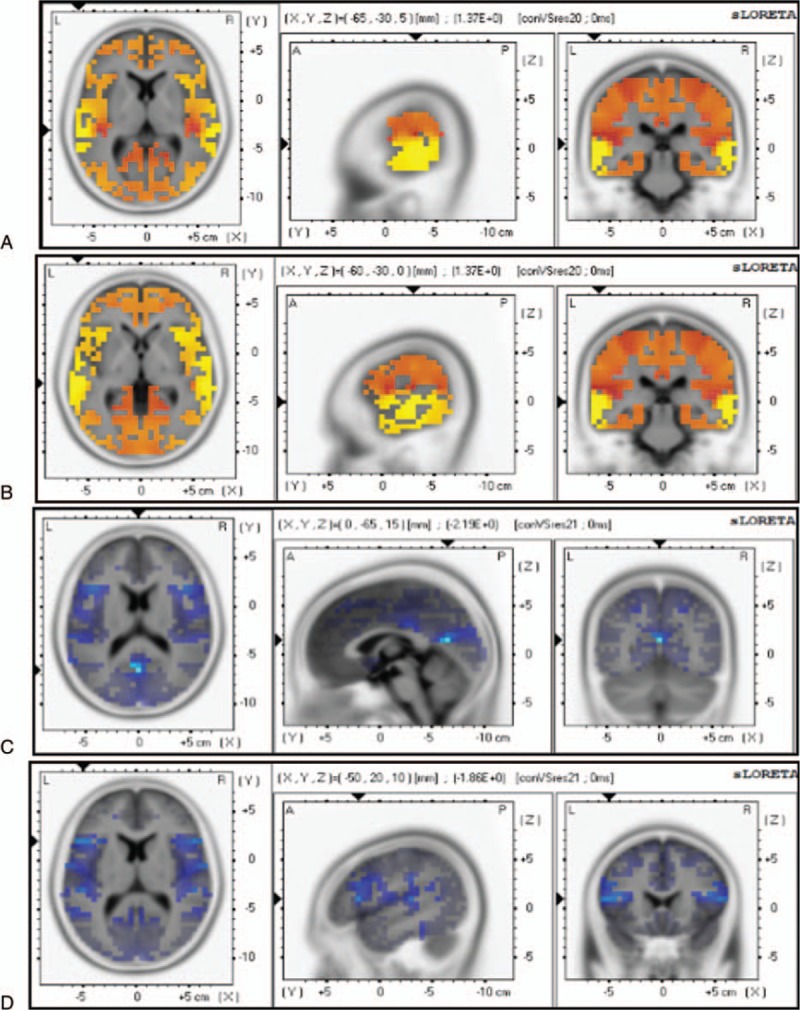
sLORETA images focusing on the largest difference observed between the healthy controls and the OCD patients in the flanker task (Experiment II). Yellow-red indicates the activated areas between the 2 groups, and blue indicates a significant decrease of sLORETA values: (A). Superior temporal gyrus (x, y, z = 65, −30, 5; BA = 42); (B). Middle temporal gyrus (x, y, z = −60, −30, 0; BA = 21); (C). Posterior cingulate (x, y, z = 0, −65, 15; BA = 23); (D). Inferior frontal gyrus (x, y, z = −50, 20, 10; BA = 45). *P* < 0.05. OCD = obsessive-compulsive disorder, sLORETA = standard low-resolution electromagnetic tomography.

Statistical comparative analysis of the visual ERP waveforms (latency range: 50–150 ms after target onset) showed significant differences between the healthy controls and the responding OCD patient subgroup (Fig. 7A and B). Specifically, the responding OCD patient subgroup had decreasing current densities in the superior/middle temporal gyrus, but increasing current densities in the posterior cingulate and interior frontal gyrus (Fig. 7C and D and Table 7). There were no significant differences between the refractory and responding OCD patient subgroups.

**Table 7:**
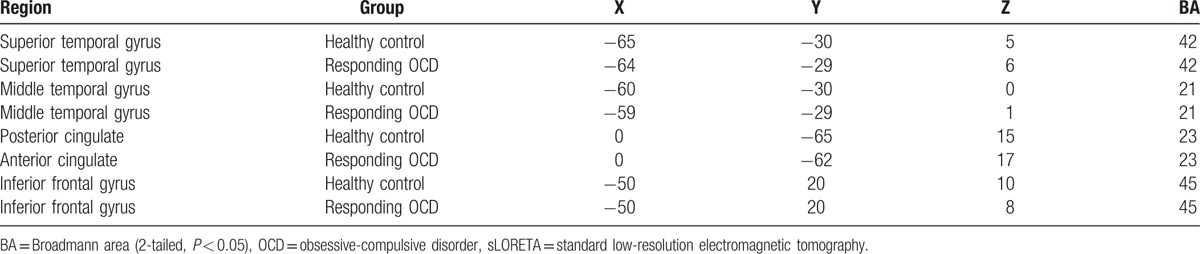
Significant differences between the healthy controls and the OCD patients preceded in sLORETA in the flanker task.

## 4 Discussion

In the present study, the visual search task was designed to further understand visual cognition dysfunction in patients with OCD. Refractory OCD patients had significantly prolonged latencies, increased numbers of saccades, and prolonged fixation durations than the healthy controls. There was no significant difference between the healthy controls and the responding subgroup. Patients with OCD were more likely to be distracted by irrelevant thoughts and movements, and there was a clear relationship between therapeutic responsiveness and improvements in visual cognitive ability.

Furthermore, we evaluated the relationship between electrophysiological patterns and the visual search task. The components (components 1 and 2) of the visual ERPs in the visual search task varied with some well-known components of ERPs, such as N1, N2, and P300.^[22]^ The refractory OCD subgroup had a significantly longer latency for component 1, compared to both the healthy controls and the responding OCD subgroup. According to the scalp map topography data and the latency difference, component 1 was likely triggered by the ACC system, since the scalp EEG was not substantially projected on central areas of the brain. The ACC is part of the cingulate cortex and is located on the medial surface of the frontal lobe and consists of Brodmann areas 24, 32, and 33.^[29]^ In addition, analysis of significantly different waveforms at electrodes C3 and C4 between patients with OCD and healthy controls revealed that component 1 was closer to the parietal lobe. Therefore, we deduced that Brodmann area 24 was the source of the component. The refractory OCD subgroup showed a lower-peak amplitude in component 2, compared to both the healthy controls and the responding OCD subgroup. Furthermore, sLORETA analysis showed that compared to the responding OCD patients, the refractory OCD patients showed higher activation in Brodmann area 40, which was located in the fusiform gyrus between the inferior temporal gyrus and the par hippocampal gyrus. Functionally, the fusiform gyrus is important for the processing of color information, face and body recognition, and word recognition.^[30,31]^ The refractory OCD patients had more saccades and a longer fixation duration time, consistent with serious dysfunctions in facial feature recognition. The visual word form area is a functional region in the middle part of the fusiform gyrus that is hypothesized to be involved in lower-level word identification, before association with phonology or semantics.^[32]^ That the amplitude of component 2 was decreased in the refractory OCD patients indicated an early word processing disorder among them. Thus, these patients failed to distinguish between word graphemes and meanings during the visual search trials, resulting in a prolonged saccade latency, a decreased accuracy rate period, and a longer response time.

In this study, we also evaluated the relationship between error-detection ERPs and cognitive and executive function in OCD patients using a modified lateral conflict task. The flanker task is often used in ERN research o generate greater conflict and higher error rates, thus resulting in stable ERN compositions. We found a significant difference in accuracy rates only between the healthy controls and the refractory OCD subgroup. Compared to the most similar flanker task, the accuracy rates in the present study were similar in the healthy controls, whereas they were significantly lower in the OCD patients.^[7]^ This difference indicated that the use of the modified flanker task may reveal some pathological characteristics of OCD, as the refractory OCD patients showed more stable symptoms and a lower sensitivity to treatment.

Inconsistent with previous studies showing that ERN amplitudes are enhanced in OCD patients, the refractory OCD subgroup showed significantly smaller ERN amplitudes than the healthy controls, with the largest ERN peak amplitudes appearing mainly on the forehead position in the present study.^[33–37]^

At present, there are 2 hypotheses about the origin of ERN: ACC origin and DLPFC origin. The ACC is involved in the fronto-striatal-thalamic-cortical circuits.^[38]^ ERN studies have shown that the ACC appears to receive information about a stimulus, select an appropriate response, monitor the action, and adapt the behavior in the presence of a conflict.^[39]^ In OCD, some patients have an elevated level of glutamate in the ACC, consistent with excessive activity in that area.^[40]^ DLPFC consists of Brodmann areas 9 and 46 and is important for the performance of executive functions, such as working memory, cognitive flexibility, planning, inhibition, and abstract reasoning. The ERN of subjects with a high level of distractibility is thought to be generated in the DLPFC.^[41]^ Consistent with previous studies, the ERN amplitudes in the present study were small in other regions except for the projection areas of the ACC and DLPFC. Sanz et al^[42]^ have reported that the P300 component of the ERP has a lower amplitude and a longer latency in OCD patients when compared with controls, and cognitive dysfunction and compulsive behaviors in OCD patients have been related to abnormalities within the DLPFC. As localization of DLPFC has been suggested to be closer to the frontal lobe than the ACC,^[43]^ we believe that the ERN measured at electrodes Fz and FPz, which exhibited similar latencies as P300, was likely because of activity in the DLPFC. In addition, executive dysfunction of the refractory OCD subgroup may be related to the inhibitory activity indicated by the presence of overactive performance monitoring.

It is generally believed that both cultural and genetic factors determine the variations in OCD pathophysiology among different populations.^[44]^ For example, Turkish patients are more likely to exhibit hoarding or aggression syndrome,^[45]^ whereas Japanese patients are more likely to present with contamination/washing syndrome.^[46]^ Although it has been proposed in North American patients that a functional polymorphism of the catechol-o-methyl transferase gene may play a role in the pathogenesis of OCD, a subsequent study in Japan did not confirm this result.^[47]^ Despite this finding, it does appear that the manifestation of OCD and other cognitive symptoms involving blasphemous thoughts or impulse control depends on various cultural contexts.^[48]^ Based on our findings, we hypothesize that population diversity in ERN generation may cause variable cognitive dysfunction in patients with refractory OCD.

## 5 Conclusion

We demonstrated that cognitive and executive function, including nonverbal memory, visuospatial skill, visual attention, and selective attention, are significantly impaired in patients with refractory OCD. The refractory OCD subgroup presented with distractibility and insufficient inhibitory activity, which we attributed to abnormal activity within the fusiform gyrus and DLPFC. Taken together, our findings suggest that dysregulation of these areas plays important roles in the pathophysiology and refractoriness of OCD. Further studies will allow for optimization and refinement of treatment strategies for refractory OCD.
